# Disparities in diabetes and sedentary behavior across Florida counties

**DOI:** 10.34172/hpp.43402

**Published:** 2024-12-30

**Authors:** Dottington Fullwood, Justine Gunderson, Opeyemi O. Bolajoko, Randy Hale, Folakemi T. Odedina

**Affiliations:** ^1^Cancer Prevention, Survivorship and Care Delivery (CPSCD) Research Program, Mayo Clinic Comprehensive Cancer Center, Jacksonville, Florida, USA; ^2^Policy and Regulatory Science Program, RTI International, Social Statistical and Environmental Sciences Unit, Research Triangle Park, North Carolina, USA; ^3^iCCaRE for Black Men Consortium, Mayo Clinic, Jacksonville, Florida, USA

**Keywords:** Diabetes, Health disparities, Florida geography, Rurality, Sedentary behavior

## Abstract

**Background:**

This study aimed to analyze the geographic distribution of diabetes and sedentary behavior across Florida counites, identifying high-risk areas to inform targeted public health interventions. We seek to inform targeted public health interventions and address health disparities, particularly in rural areas.

**Methods:**

Data from the 2017-2019 Florida Behavioral Risk Factor Surveillance System (BRFSS) were analyzed, among adults aged 18 years and older. Diabetes status was determined by self-reported diagnosis, and sedentary behavior was assessed based on physical activity participation. Respondents who declined to answer or were unsure were excluded.

**Results:**

The statewide prevalence of diabetes diagnosis in Florida was 11.7% (95% CI: 10.8-12.6), with substantial geographic variation across counties. Sedentary prevalence varied significantly across the state, compared to the statewide rate of 26.5% (95% CI: 25-28). Counties with high diabetes prevalence often had elevated rates of sedentary behavior. Rural northern panhandle counties had higher concentrations of both diabetes and sedentary behavior. BRFSS design variables and weights ensured representative estimates for Florida.

**Conclusion:**

This study highlights the elevated prevalence of diabetes and sedentary lifestyles in the northern counties of Florida. The geographic patterns underscore the need for targeted, county-level interventions. Despite troubling rise in diabetes and sedentary behavior, there is a significant opportunity to mobilized community and outreach services, ensuring they are effectively deployed to these high-risk areas.

## Introduction

 Diabetes and a sedentary lifestyle are health issues that may occur independently or together, resulting in decreased life expectancy.^1^ Research links sedentary lifestyle to abdominal obesity and impaired insulin, which increases the risk of diabetes and poor outcomes.^2^ With over 38.4 million people with diabetes—representing 11.6% of the population^3^—the prevalence of diabetes is a public health concern and growing epidemic in the United States.

 Florida is considered part of the “diabetes belt,^4^” a geographic region in United States with clustered patterns of higher-than-average diabetes prevalence that spans across 15 Southern states. For years, Florida has consistently ranked higher than the national average for diabetes prevalence.^5^ the adult diagnosis rate was approximately 13.1%, with another 9.9% of adult Floridians diagnosed with prediabetes.^6,7^ Among Black Americans over the age of 18, 12.2% were diagnosed with diabetes in 2018-2019, and the mortality rate for Black men in 2019 was 47.1%.^8^Unfortunately, almost 550 000 Floridians are unaware they have diabetes,^7^ and the consequences including increased medical expenses and indirect cost from the inability to work, further compound the negative health outcomes.

 Diabetes is more prevalent among rural populations compared to their urban counterparts, and managing the condition is particularly challenging due to the need for lifelong care.^9,10^ Evidence indicates significant disparities in diabetes control and outcomes among rural dwellers across various countries.^11-13^ These healthcare disparities between rural and urban dwellers have been well-documented. Studies reveal that access to care is often limited in rural areas, and rural residents may experience higher diabetes mortality rates than those in urban settings. Various social determinants, such as long distances to health facilities and lack of access to specialty care, exercise facilities, internet, and efficient telecommunication, are known to impact diabetes management in rural settings. Consequently, living in rural areas not only hampers healthcare access but also promotes sedentary lifestyles due to the lack of exercise facilities. A sedentary lifestyle is a known risk factor for the onset of type 2 diabetes and adversely affects blood glucose control.^14^

 Defining the specific geographic distributions of health issues for interventional programs and research is important for investigators, practitioners, and policymakers to inform appropriate resource allocation and priority areas for targeted interventions.^15^ While other studies have investigated the geographic distribution of diabetes at the state level,^16^ few studies have explored more granular spatial patterns between diabetes prevalence and race/ethnicity to identify diabetes hotspots, provide evidence for appropriate interventions, and combat disparities in diabetes prevalence.

## Methods

 This study aimed to define the geographical distribution of all Floridians who reported having diabetes between 2017-2019 and understand how this relates to sedentary behavior in Florida.

###  Study design

 Sample survey data from the Florida Behavioral Risk Factor Surveillance System (BRFSS) was retrieved from civilian, noninstitutionalized US adults aged 18 years or older. The annual survey is distributed via random-digit-dialed landlines and cell phones. We analyzed data on diabetes and sedentary behavior from the 2017-2019 county-level BRFSS.^17^

###  Data collection 

 Diabetes status was assessed by the answer to the question, “*Have you ever been told by a doctor, nurse, or other health professional that you have diabetes?*” Individuals who answered “No” to the question “*During the past month, other than your regular job, did you participate in any physical activities such as running, calisthenics, golf, gardening, or walking for exercise?*” were classified as sedentary. We excluded those who declined to answer these questions or reported “not sure” or “don’t know.”

###  Data analysis

 All analyses were conducted using appropriate BRFSS design variables and weights, ensuring estimates were representative of the Florida population. SAS version 9.4 (Cary, NC: SAS Institute Inc) was used to calculate the overall prevalence of diabetes cases across Florida and determine the weighted mean of diabetes across the 67 counties.

## Results

 While the statewide prevalence of diabetes diagnosis was 11.7% (95% CI: 10.8–12.6), the geographic distribution of diabetes prevalence varied substantially across counties in Florida, ranging from a high of 20.8% (95% CI: 14–27.6) in Glades County to a low of 6.4% (95% CI: 4.5–8.3) in Leon County. Many counties in the central region of the state tended to have higher diabetes prevalence compared to southern coastal counties. Additionally, higher diabetes prevalence was observed in some northern panhandle counties near the Florida-Georgia border.

 The prevalence of sedentary behavior also varied widely across the state, compared with the statewide rate of 26.5% (95% CI: 25–28) Prevalence of sedentary behavior was ranked by county and ranged from a high of 42.7% (95% CI: 35.5–49.9) in Hardee County to a low of 19.3% (95% CI: 14.9–23.8) in Leon County (Table 1). More than half of Florida counties had a prevalence rate of sedentary behavior greater than 30%. Many of the counties with high diabetes prevalence also had elevated rates of sedentary behavior. Some of the counties with both a high prevalence of diabetes and sedentary behavior were located in rural northern panhandle counties. For example, in Gadsden County, a county where 38.9% (95% CI: 29.6–48.3) of the population was sedentary, nearly 20% (19.9%) reported a diabetes diagnosis. Similarly, in neighboring Holmes County, 40.0% (95% CI: 34.3–45.6) of respondents were sedentary and 18.4% had diabetes.

**Table 1:** Ranked prevalence (%) of self-reported sedentary behavior* among adults aged ≥ 18 years by county, Florida Behavioral Risk Factor Surveillance System, 2017-2019

	**Rank**	**Weighted %**	**95% CI**	
Florida		26.5%	25.0%	28.0%
Hardee	1	42.7%	35.5%	49.9%
Hendry	2	42.0%	35.9%	48.2%
Levy	3	40.2%	34.2%	46.2%
Holmes	4	40.0%	34.3%	45.6%
Lafayette	5	39.5%	29.9%	49.2%
Gadsden	6	38.9%	29.6%	48.3%
Calhoun	7	38.7%	33.2%	44.1%
Dixie	8	38.1%	32.7%	43.5%
Okeechobee	9	37.7%	32.1%	43.2%
Glades	10	36.8%	28.1%	45.4%
Leon	67	19.3%	14.9%	23.8%
Sumter	66	21.5%	16.6%	26.4%
Alachua	65	21.8%	17.8%	25.8%
Pinellas	64	22.0%	18.4%	25.5%
Sarasota	63	22.0%	18.0%	26.1%
St. Johns	62	22.2%	18.4%	26.0%
Seminole	61	22.6%	18.4%	26.8%
Monroe	60	23.2%	17.9%	28.6%
Broward	59	24.1%	20.7%	27.4%
Flagler	58	24.7%	20.3%	29.0%

Abbreviation: CI, confidence interval. *Sedentary behavior is defined as no physical activity or exercise during the past 30 days outside of job duties.

## Discussion

 These findings suggest that many Florida counties have both an elevated prevalence of diabetes and sedentary behavior, particularly in more rural areas of the state. This trend will negatively impact health outcomes in these populations by increasing their risk of other chronic diseases such as cancers. For men who have diabetes, androgen levels tend to be moderately reduced, and the interaction between metabolic and androgen factors may increase their risk of prostate cancer.^2^ This might contribute to the disparities in prostate cancer prevalence and increased mortality risk observed in Black men.^18^

 The higher prevalence of diabetes and sedentary lifestyle observed in the northern panhandle counties could impact productivity and economic status, further enforcing health disparities within the underserved and rural communities. Compounding these effects, research shows, an inverse correlation was found between sedentary behavior and socioeconomic factors among older adults, and status of health improves with increased social advantage.^19^

 Identifying these regions in Florida at increased risk for diabetes and sedentary behaviors is critical for deploying targeted interventions and improving access to care for vulnerable populations. This vivid snapshot unveils a growing menace of health disparities gripping rural communities predominantly inhabited by individuals who experience lack of healthcare access, resulting in poorer health outcomes.

 Several limitations should be noted related to survey methodology and design. First, BRFSS data are cross-sectional, which limits our ability to determine causal pathways for diabetes risk. Second, BRFSS data are self-reported. Although research has demonstrated alignment between self-reported diabetes diagnosis and externally verified diagnosis of diabetes,^20^ our results could underestimate the true prevalence of diabetes in the population due to the inability to capture undiagnosed diabetes. Sedentary behavior may also be subject to recall and social desirability bias, which may result in an underestimate of sedendary behavior. Lastly, BRFSS design excludes the institutionalized population and findings may not be generalizable to this population. Despite these limitations, our study has substantial strengths in its ability to leverage county-level data to pinpoint geographic areas with elevated risk for diabetes and sedentary behavior across the state.

## Conclusion

 This study reveals that higher diabetes and sedentary lifestyles may be more concentrated in the northern part of Florida counties. Our mapping provides initial evidence that may bolster support for county-specific level interventions for high-prevalence counties, leading to more precise identification of high-risk populations. In spite of the disconcerting surge of diabetes and sedentary behavior incidence in tandem, there lies an immense opportunity to mobilize community and outreach services to more effectively target diabetes management and prevention efforts, as well as physical activity interventions, ensuring their timely deployment to these vulnerable landscapes.

## Competing Interests

 The authors have no funding or conflicts of interest to disclose.

## Ethical Approval

 This study was performed in line with the principles of the Declaration of Helsinki. Approval was granted by the Mayo Clinic Florida Institutional Review Board (IRB#23-000375).
